# Plant Functional Group Removal Shifts Soil Nematode Community and Decreases Soil Particulate Organic Carbon in an Alpine Meadow

**DOI:** 10.3390/plants14243728

**Published:** 2025-12-06

**Authors:** Ligai Huang, Luping Ye, Xianhui Zhou, Hui Guo, Juan Zuo, Peng Wang, Yong Zheng

**Affiliations:** 1 Hunan Cultivated Land and Agricultural Eco-Environment Institute, Changsha 410125, China; ligaihuang25@126.com; 2 Wuhan Botanical Garden, Chinese Academy of Sciences, Wuhan 430074, China; 3 Danjiangkou Wetland Ecosystem Field Scientific Observation and Research Station, Chinese Academy of Sciences & Hubei Province, Wuhan 430074, China; 4 State Key Laboratory of Grassland and Agroecosystems, College of Ecology, Lanzhou University, Lanzhou 730000, China; 5 College of Resources and Environmental Sciences, Nanjing Agricultural University, Nanjing 210095, China

**Keywords:** plant functional groups, plant species loss, soil carbon fractions, soil nematode community, soil food web

## Abstract

Vegetation degradation in the alpine meadows is becoming increasingly severe under global change, with species loss frequently linked to changes in plant functional groups (PFGs). Changes in PFGs alter plant-derived carbon inputs, which significantly influence soil organic carbon (SOC) sequestration and soil communities. However, the impact of specific PFG removal on soil carbon fractions and nematode trophic groups remains underexplored. In this study, above-ground removal of PFGs was carried out for five consecutive years in the Qinghai–Tibet Plateau alpine meadow, with five treatments: (1) no removal of PFGs (CK); (2) keep non-legume forbs (remove graminoids and legumes, Forbs); (3) keep graminoids (remove legumes and non-legume forbs, Graminoids); (4) keep legumes (remove non-legume forbs and graminoids, Legumes); (5) remove all PFGs (All-plants-removed). Root properties, nematode community, and soil carbon fractions were measured. We found that the Graminoids treatment significantly increased root biomass, whereas the All-plants-removed treatment led to a significant decrease. Nematode abundance was highest under the Legumes treatment, primarily due to increased omnivores-predators. Meanwhile, the soil particulate organic carbon (POC) varied significantly between PFG types, being the highest in the Forbs and CK treatments. Correlation analysis revealed a significant positive relationship between SOC and bacterivore abundance, suggesting that higher SOC enhances bacterivore populations and subsequently influences carbon cycling. We conclude that PFG removal alters soil nematode community structure and POC, underscoring the role of PFGs in below-ground biodiversity and soil carbon sequestration.

## 1. Introduction

Understanding the consequences of plant species loss on ecosystem functioning and services has drawn considerable attention in the last two decades [1,2,3]. The ongoing trend of plant functional diversity loss is primarily driven by global changes and human activities [4,5]. For example, warming has reduced forb and graminoid diversity [6], and selective grazing by livestock has decreased graminoid abundance [7]. Additionally, fertilization has led to forb loss due to their lower competitive ability for nutrients and light [8]. Numerous studies in artificially assembled communities have shown that functional group diversity loss strongly impacts ecosystem functioning and services, with diminishing returns at higher diversity levels [9,10]. These studies highlight the impacts of PFG loss, but their findings often focus on the functioning of above-ground ecosystem components. In fact, shifts in PFGs also exert significant influence on below-ground community structure and soil processes [11,12,13]. However, the mechanisms by which PFG losses affect below-ground communities and their linkages with soil carbon sequestration remain poorly understood.

In natural alpine meadow ecosystems, plant species are typically classified into forbs, graminoids, and legumes based on resource use strategies [5,14]. These PFGs differ in their contributions to soil processes, with each PFG influencing below-ground communities and carbon dynamics in unique pathways. Legumes enhance nitrogen availability through atmospheric fixation and release high-quality organic matter, improving soil fertility and soil food web complexity [15,16,17,18]. However, the ecological importance of legumes may be limited by their low biomass, typically less than 5% of total biomass in alpine meadows. In contrast, the mass ratio hypothesis predicts that ecosystem properties are primarily driven by the traits of dominant species [19,20,21]. In alpine meadows, where forbs and graminoids dominate biomass, their loss may have greater impacts on soil processes than the less abundant legumes. Thus, further research is necessary to clarify how the loss of legumes versus dominant forbs and graminoids affects below-ground communities and soil processes, such as carbon storage.

Soil nematodes occupy multiple trophic levels and are classified into functional guilds (bacterivores, fungivores, plant parasites, omnivores-predators) based on feeding traits [22,23,24]. Nematode abundance and community composition are highly sensitive to changes in the above-ground plant diversity [25,26,27,28]. For instance, graminoid removal suppressed plant-parasitic nematodes [29], while shrub removal decreased bacterivores, fungivores, and plant parasites in biodiversity experiments [12]. Long-term graminoid removal increased bacterivores but reduced plant parasites, with opposite effects from forb removal [30]. These changes in nematode community are closely tied to plant-derived carbon inputs, which also influence soil carbon dynamics.

Plant-derived carbon supports below-ground soil communities across trophic levels and serves as a major source of soil organic carbon (SOC) [31,32,33]. A grassland biodiversity experiment showed that SOC increased with plant species richness [34], while other studies suggested that the increase in organic carbon accumulation is largely driven by the presence of specific functional groups. Fornara and Tilman [35] attributed the increase in soil carbon sequestration to the presence and abundance of legumes and C4 grasses in North American tallgrass prairies. In contrast, Li et al. [13] found that compared to forbs, the above-ground biomass of graminoids was significantly negatively correlated with changes in SOC.

The Qinghai–Tibetan alpine meadow is a unique ecosystem with a diverse flora in high-altitude regions. The humid and cold plateau climate with an average annual temperature of 2 °C and with more than 250 frost days per year contributes to the accumulation of SOC. This high SOC supports diverse soil microbial communities, which in turn influence nematode abundance and trophic structure [36,37,38]. For instance, Zhao et al. [39] found that changes in organic carbon caused by legumes were a key factor influencing soil nematode community; SOC was correlated positively with omnivores-predators density and the nematode maturity index (MI) [40]. Among different nematode trophic groups, soil organic carbon is beneficial to the abundance of omnivores-predators, but it is not conducive to the abundance of bacterivores, fungivores, and plant parasites [41]. Particulate organic carbon (POC) and mineral-associated organic carbon (MAOC) are considered essential for understanding the dynamics of SOC under climate change [42,43]. Plant carbon input is one of the main factors affecting the changes in POC and MAOC [43,44]. Therefore, shifts in the composition of PFGs with different nutrient utilization strategies and plant traits may lead to changes in soil carbon fractions, which in turn influence nematode community structure. Therefore, a comprehensive understanding of how a reduction in PFG richness affects below-ground communities and their linkages to C storage in natural ecosystems needs to be further clarified.

After 5 years of consecutively removing specific PFGs in a Qinghai–Tibet Alpine meadow, we investigated the effects of PFG removal on soil nematodes and soil carbon fractions. We hypothesize that: (1) PFG removal will reduce nematode abundance, with PFG identities shaping community structure. Specifically, legumes will increase bacterivore abundance due to enhanced microbial activity from nitrogen-rich root exudates, while graminoids will promote plant-parasitic nematodes due to their extensive root systems. (2) PFG identities affect soil carbon fractions through root properties. Legumes, with high-quality roots, will enhance SOC accumulation, supporting bacterivore populations, while graminoids, with higher below-ground biomass, will increase POC due to greater root inputs.

## 2. Results

### 2.1. PFGs Removal Effects on Root Biomass and Root Properties

**Figure 1:**
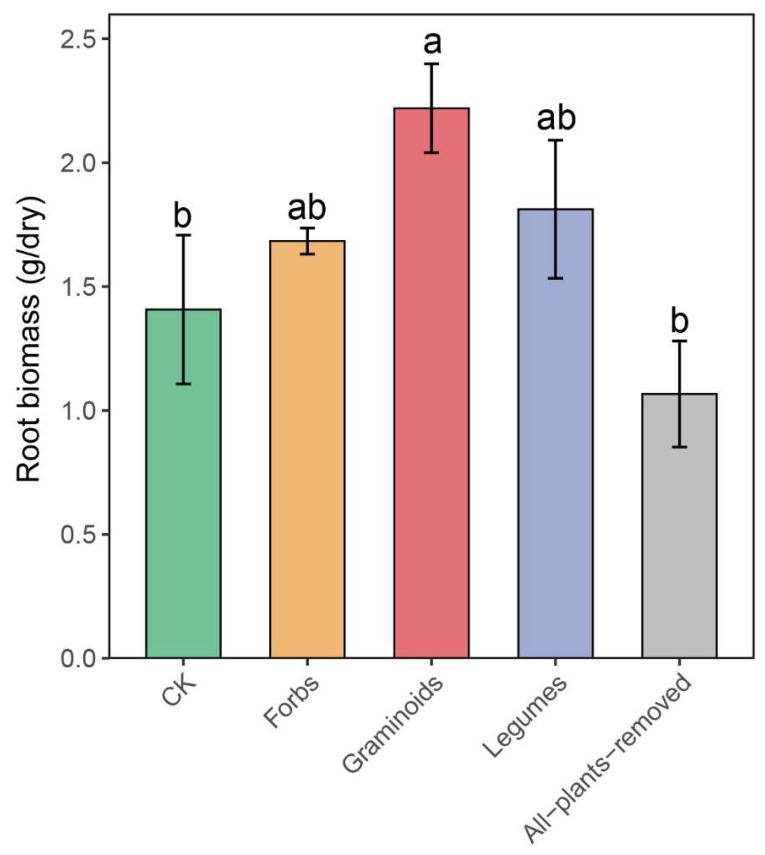
The effects of plant functional group (PFG) removals on root biomass. Bars are means ± standard errors, *n* = 5. Lowercase letters labeled above bars represent the results of multiple comparisons. g/dry indicates the root dry weight in 100 g of dry soil.

Root biomass differed significantly among the five PFGs removal treatments. Graminoids treatment had the highest root biomass, while CK and All-plants-removed treatments had the lowest (*F* = 4.716, *p* = 0.006; Figure 1; Table 1). Compared to the CK treatment, the Forbs, Graminoids, and Legumes treatments led to a 19.15%, 57.45%, and 28.37% increase in the root biomass, respectively. Root total carbon, root total nitrogen, and root carbon-to-nitrogen ratio did not differ significantly between the treatments (Table 1).

### 2.2. PFGs Removal Effects on Soil Nematode Community

**Figure 2:**
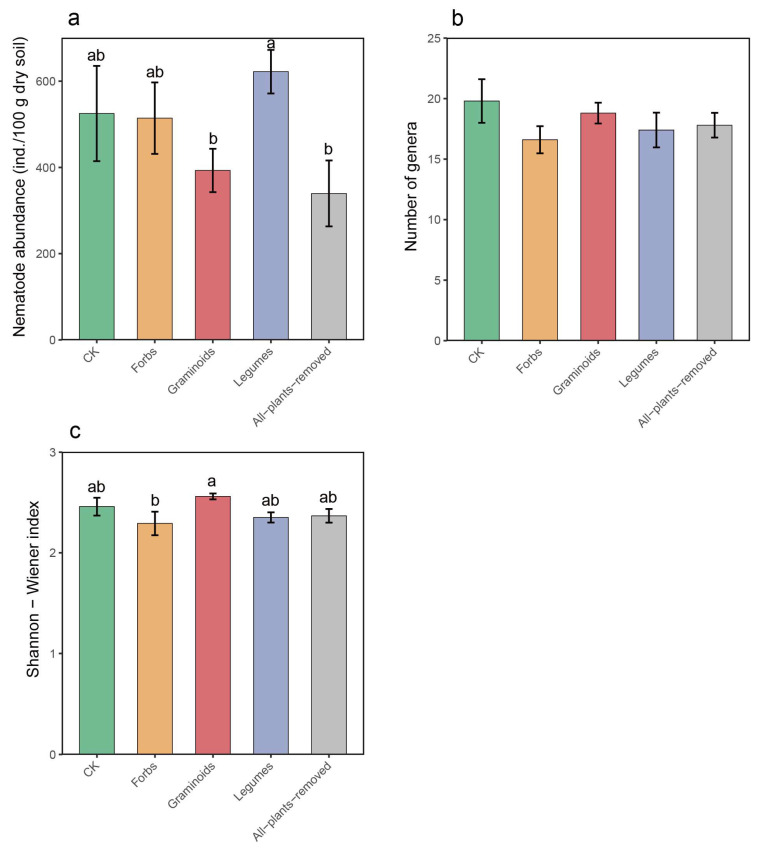
Effects of plant functional group (PFG) removals on nematode abundance (**a**), nematode richness (**b**), Shannon–Wiener index (**c**). Bars are means ± standard errors, n = 5. Lowercase letters labeled above bars represent the results of multiple comparisons.

A total of 11,969 nematode individuals were recorded, representing 36 genera. The total abundance of nematodes was significantly affected by PFG removals (*F* = 4.395, *p* = 0.011; Figure 2a; Table 2). The highest total abundance of nematodes was found in the Legumes treatment (622 individuals per 100 g dry soil), and the lowest was found in the All-plants-removed treatment (340 individuals per 100 g dry soil). The Shannon diversity index (H’) of the nematode community also differed significantly among PFG removals; the Graminoids treatment had the highest, and the Forbs treatment had the lowest diversity (*F* = 3.321, *p* = 0.031; Figure 2c; Table 2). Nematode richness at the genus level did not differ among PFG removals (*p* > 0.05; Figure 2b).

PFG removals had significant effects (*F* = 5.933, *p* < 0.05; *F* = 27.475, *p* < 0.01; *F* = 7.392, *p* < 0.01) on SI, MI, and PPI, but no significant effect on NCR, CI, and EI (Table 2). The structure index (SI) in the Graminoids treatment was significantly lower than in the other four treatments. The Forbs and Graminoids treatments led to a −15.67% and −18.81% decrease in the MI, respectively, compared to the CK treatment. The plant parasite index (PPI) was significantly higher (*F* = 7.392, *p* < 0.001) in the Forbs treatment (0.87), followed by the Graminoids (0.55), Legumes (0.53), CK (0.48), and All-plants-removed treatments (0.40). There was a linear trend of increase in PPI following the Forbs treatment, whereas there was a linear decrease in MI. The Forbs treatment had the highest CI (75.20), while the lowest CI was observed in the All-plants-removed treatment (30.87) (Table S1).

**Figure 3:**
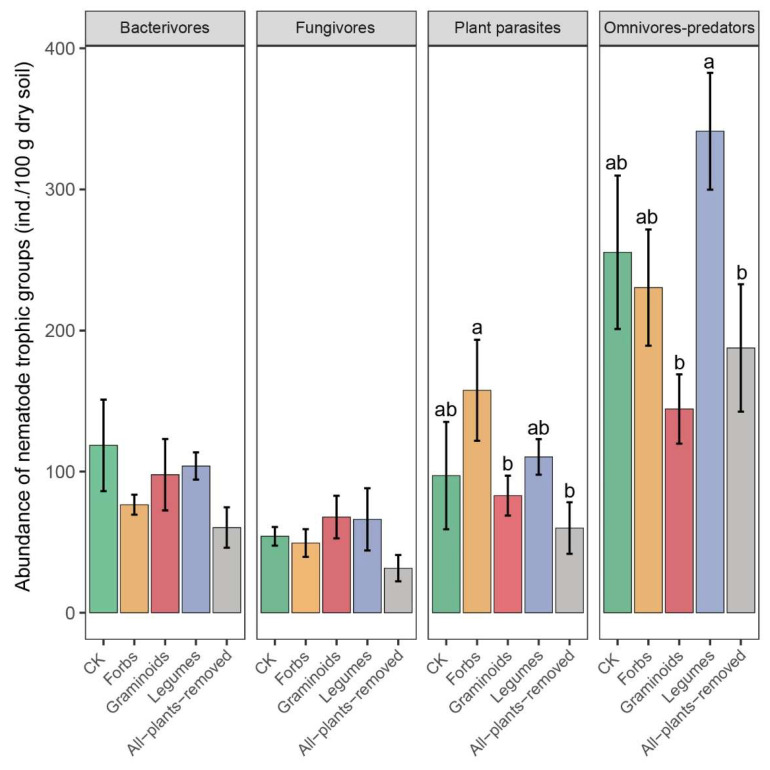
The effects of plant functional group (PFG) removals on nematode trophic groups abundance. Bars are means ± standard errors, n = 5. Lowercase letters labeled above bars represent the results of multiple comparisons.

The nematode community was dominated by omnivores-predators, followed by plant parasites, bacterivores, and fungivores. The nematode community was dominated by omnivores-predators, followed by plant parasites, bacterivores, and fungivores (Figure 3; Table 3). PFG removals had a significant effect (*F* = 3.573, *p* < 0.05; *F* = 3.890, *p* < 0.05) on the plant parasites and omnivores-predators (Table 2). The Forbs treatment had the highest abundance of plant parasites, while the All-plants-removed and Graminoids treatments had the lowest abundance of plant parasites; the Legumes treatment had the highest abundance of omnivores-predators, while the Graminoids and All-plants-removed treatments had the lowest abundance of omnivores-predators (Figure 3).

### 2.3. Linkage Between Soil Carbon Fractions and Nematode Community Under PFGs Removal

**Figure 4:**
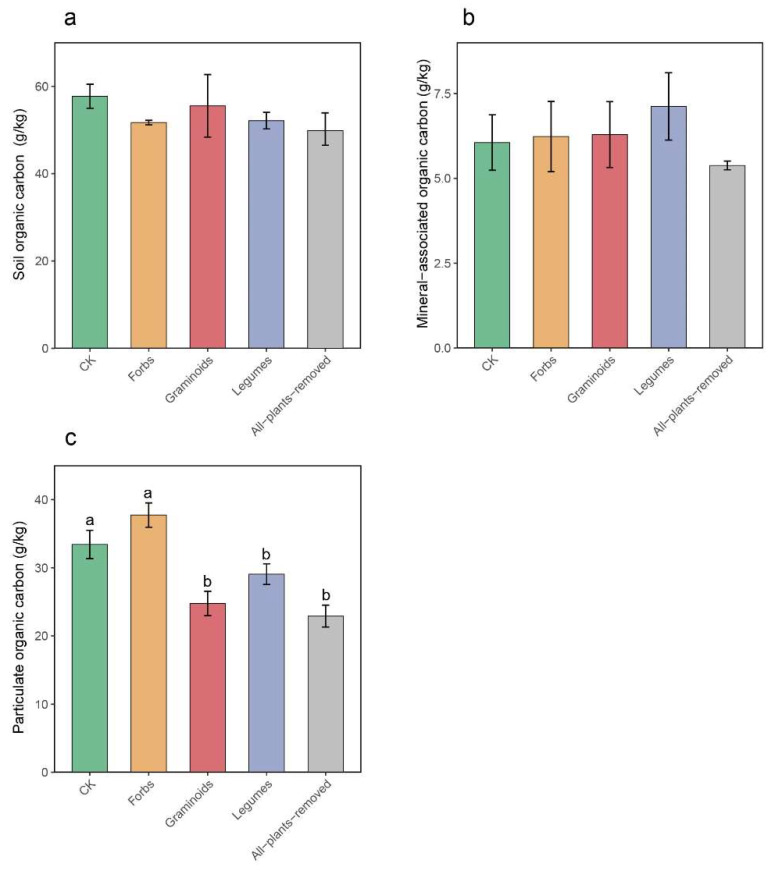
Effects of plant functional group (PFG) removals on soil organic carbon (**a**), mineral-associated organic carbon (**b**), and particulate organic carbon (**c**). Bars are means ± standard errors, n = 5. Lowercase letters labeled above bars represent the results of multiple comparisons.

PFG removals had a significant effect (*F* = 14.972, *p* < 0.01) on particulate organic carbon (POC) but no significant effect on soil organic carbon (SOC) and mineral-associated organic carbon (MAOC) (Figure 4; Table 1). The POC in the Forbs and CK treatments (37.74 and 33.42 g kg^−1^) was significantly higher than that in the Legumes, Graminoids, and All-plants-removed treatments (29.06, 24.76, and 22.91 g kg^−1^, respectively) (Table S2).

**Figure 5:**
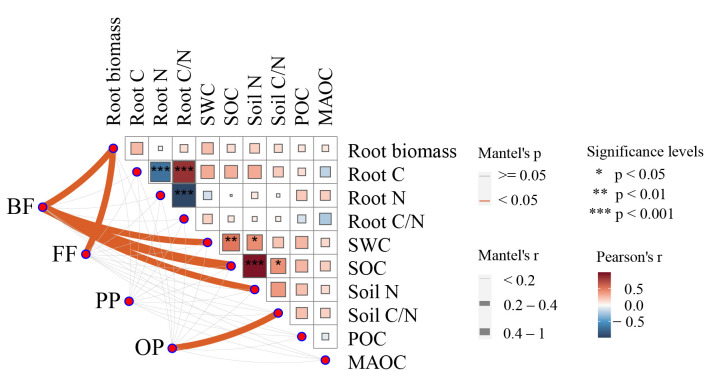
Environmental drivers of nematode community composition. Pairwise comparisons of environmental factors are shown, with a color gradient denoting Pearson’s correlation coefficients. Nematode trophic groups (bacterivores, BF; fungivores, FF; plant parasites, PP; omnivores-predators, OP) were related to each environmental factor by partial Mantel tests. Edge width corresponds to Mantel’s r statistic for the corresponding distance correlations, and edge color denotes the statistical significance based on 9999 permutations.

The Mantel test revealed that the soil nematode community was strongly associated with plant root and soil environmental factors (Figure 5). The bacterivores were strongly positively correlated with root biomass (Mantel’s r > 0.2, *p* < 0.05), SWC (Mantel’s r > 0.2, *p* < 0.05), SOC (Mantel’s r > 0.4, *p* < 0.05), and soil nitrogen (Mantel’s r > 0.2, *p* < 0.05). The fungivores were strongly positively correlated with root biomass (Mantel’s r > 0.2, *p* < 0.05). The omnivores-predators were positively correlated with the soil carbon to nitrogen ratio (Mantel’s r > 0.2, *p* < 0.05).

## 3. Discussion

### 3.1. Effects of PFG Removal on Root Properties

Our results showed that the Graminoids treatment significantly enhanced root biomass, which is consistent with previous studies [45,46,47]. This is probably due to the removal of other PFGs reduced resource competition for the dominant graminoid species, therefore leading to an increase in below-ground root biomass [48]. Moreover, we observed that while legumes contributed only 1% to above-ground biomass, their below-ground biomass showed an increasing trend following the removal of other PFGs. Two mechanisms may explain the increased allocation of below-ground biomass by legumes. First, in multispecies plant communities, legumes typically reduce above-ground competition for light and allocate more biomass to their roots. This shift in allocation transfers competitive pressure to below-ground organs, allowing legumes to better access soil resources [49]. Second, legumes promote root growth to support their symbiotic relationship with rhizobia, enhancing nitrogen fixation efficiency [50]. We observed that root biomass in the CK treatment had a trend of higher than in the All-plants-removed treatment, while this difference was not statistically significant. This is likely because of residual roots remaining in the soil and root regeneration.

### 3.2. Effects of PFG Removal on Soil Nematode Community

PFG removals significantly affected nematode abundance, with the Legumes treatment showing higher abundance than the Graminoids and All-plants-removed treatments, supporting our first hypothesis. Previous research has demonstrated that legumes primarily influence total nematode abundance through bottom-up control mechanisms, as the presence of legumes can enhance soil microbial abundance and diversity through root exudates [18,51,52]. Nematode abundance in the CK treatment was 525 individuals per 100 g dry soil, 54.41% higher than the All-plants-removed treatment. This finding indicates that the removal of PFGs not only reduces above-ground biomass but also limits litter input and root turnover, thereby restricting nematode food resources and leading to a decline in nematode abundance [53,54]. In addition, the All-plants-removed treatment increased soil exposure to solar radiation and raised surface temperatures, which may have reduced microbial activity and abundance and further limited the growth of the nematode community [55,56].

In this study, the differences in nematode abundance were mainly attributed to changes in the abundance of plant parasites and omnivores-predators. In our study, omnivores-predators accounted for the largest proportion (Figure 3). This is in contrast with the findings of Lobe et al. [57] and Liu et al. [58], who reported that bacterivores were the most abundant in soils. This discrepancy may be attributed to the alpine meadow ecosystem characterized by low temperatures, which favor the survival of larger and more resilient soil nematode species [59]. Contrary to our hypothesis, the Graminoids treatment did not increase plant-parasitic nematodes; instead, the Forbs treatment showed the highest abundance. This likely reflects the high species diversity of forbs, which provide diverse root resources as subdominant PFGs [16,20,60]. Forbs, with high-nitrogen, low-C:N root systems, support more plant-parasitic nematodes [16]. Although the abundances of bacterivores and fungivores did not differ among the different treatments, the Forbs and Graminoids treatments increased the Channel Index (CI). This indicates that the decomposition pathway in the detrital food web was predominantly driven by fungivores (*Filenchus* and *Tylencholaimus)*. In contrast, the Legumes and All-plants-removed treatments reduced the CI, shifting the decomposition pathway to be dominated by bacterivores.

### 3.3. Linkage Between Nematode Community and Soil Carbon Fractions

Our results indicated a significant increase in POC in the Forbs treatment compared to the Graminoids and Legumes treatments, primarily attributed to enhanced organic carbon inputs driven by increased plant productivity and the diversity of litter [61]. Additionally, Shen et al. [62] have highlighted soil water content as a critical factor in promoting the accumulation of POC. In our study, the POC associated with Forbs was significantly higher than other plant functional groups, with forbs also exhibiting relatively high soil water content. Although MAOC was not significantly affected by PFG removal, we observed an increase in MAOC under the Legumes treatment. This is consistent with a study conducted by Canarini et al. [63], who found that the biomass of legumes enhanced the accumulation of MAOC. Litter and roots of the legumes had lower C/N ratios, which may have stimulated microbial growth and thereby promoted the formation of MAOC [64].

Our study found a significant positive correlation between the abundance of bacterivores and SOC, which is in line with a recent study showing that bacterivores were positively affected by SOC [65]. The predation of bacteria by bacterivores can activate bacterial metabolism and accelerate SOC cycling, and also reduce the soil metabolic quotient, thereby promoting SOC accumulation [66,67]. Furthermore, we observed a significant positive correlation between bacterivores and soil nitrogen. This is likely predominantly attributed to higher soil nitrogen stimulating bacterial growth and reproduction, which provides abundant food resources for bacterivores [68]. Our study also found a positive correlation between omnivores-predators and the soil carbon to nitrogen ratio. This might be because more mature communities, whose structure is closer to the climax state, tend to store more organic carbon in organism biomass, exhibit a higher organic carbon to nitrogen ratio, and support the long-term accumulation of large numbers of omnivores and predators with long life cycles [24,69]. Unlike studies reporting SOC effects on multiple nematode groups [70,71,72], we found significant SOC correlations only with bacterivores, possibly due to the alpine meadow’s high organic matter content. This suggests that temperature, rather than resource availability, may limit nematode responses in nutrient-rich alpine meadows, warranting further investigation.

## 4. Materials and Methods

### 4.1. Field Site Description

The experiment was established in the alpine meadows on the eastern edge of the Qinghai–Tibet Plateau, at the Gannan Grassland Ecosystem National Observation and Research Station in Maqu County, Gansu province, China (33°40′ N, 101°53′ E). Details about the field site were described by Wang et al. [73] and Zheng et al. [74]. The climate of the region is characterized as a typical humid and cold plateau climate with a mean annual temperature of 1.8 °C and a mean annual precipitation of 593 mm. The altitude of the field site is about 3550 m. The soil type is subalpine meadow soil, classified by the Chinese soil classification system. This study divided plants into three PFGs based on life forms: Legumes, Graminoids, and Non-legume Forbs. The dominant graminoids were *Elymus nutans* Griseb., *Stipa aliela* Keng, and *Carex capillifolia* (Decne.) S.R.Zhang. The dominant non-legume forbs were *Saussurea nigrescens* Maxim., *Anemone rivularis* Buch. Ham. and *Aster diplostephioides* (DC.) CB Clarke. The dominant legumes were *Thermopsis lanceolata* R. Br. and *Medicago archiducis-nicolai* Sirj. The biomass of graminoids, forbs, and legumes accounted for 58%, 41%, and 1% of the total biomass, respectively.

The field experiment was initiated in August 2018, and included five treatments with five replications in a Latin square design: (1) no-removal PFGs (CK); (2) keep non-legume forbs (remove graminoids and legumes, Forbs); (3) keep graminoids (remove legumes and non-legume forbs, Graminoids); (4) keep legumes (remove non-legume forbs and graminoids, Legumes); (5) remove all PFGs (All-plants-removed). A total of 25 plots (1.5 m × 1.5 m) were set up. There was a 1 m buffer zone between each plot to reduce marginal effects. Since there is a slight slope on the field, five plots at the same elevation were considered as one block. Plants were removed three to five times during each growing season, and the above-ground parts of the target functional groups were cut off at the ground level and moved out of the plot.

### 4.2. Soil Sampling

Soil samples were collected on 30 September 2022. At each plot, three soil cores were randomly taken with a diameter of 2.5 cm and a depth of 10 cm, and then pooled into one sample. A total of 50 soil samples were obtained. About 5 g of fresh soil from each sample was used to measure soil water content (SWC), and about 80 g of fresh soil was used for the extraction and identification of soil nematodes. To assess below-ground plant inputs, plant roots were also collected from the same soil samples used for nematode extraction. The soil samples were placed in an 80-mesh sieve and washed with running water until no soil particles were left. The collected root samples were placed in an envelope and dried at 65 °C to a constant weight, and then the dry root biomass was measured. The dry plant roots were ground and passed through a 100-mesh sieve, then measured for carbon and nitrogen content using an automatic organic element analyzer (Vario TOC, Germany). Similarly, the air-dried soil was ground and passed through a 100-mesh sieve, and the soil organic carbon (SOC) and nitrogen (Soil N) content were measured using an automatic organic element analyzer (Vario TOC, Germany).

### 4.3. Nematode Extraction and Identification

Soil nematodes were separated and extracted from approximately 80 g of fresh soil using the Baermann funnel method (using trays rather than funnels) [75]. Nematodes were counted under a stereomicroscope and converted into individuals/100 g dry soil. From each sample, 100 individuals were randomly selected and identified to the genus level under an optical microscope. If the total numbers were less than 100, all were identified to the genus level. Based on soil nematodes’ feeding traits, nematodes were grouped into bacterivores, fungivores, plant parasites, and omnivores-predators [76]. The indexes of nematodes were calculated as follows:

Shannon–Wiener index: H’ = −∑ Pi × ln(Pi);
where Pi is the proportion of individuals in the i-th taxon.
Nematode channel ratio: NCR = Ba/(Ba + Fu);
Enrichment index: EI = 100 × (e/(e + b));
Structure index: SI = 100 × (s/(s + b));
Channel index: CI = 100 × 0.8Fu_2_/(0.8Fu_2_ + 3.2Ba_1_);
Free-living nematode maturity index (Plant parasite index): MI (PPI) = ∑ υ(i) × f(i).

where Ba and Fu represent the relative abundance of bacterivores and fungivores, respectively; e, b, and s are enriched components (Ba_1_ and Fu_2_), basic components (Ba_2_ and Fu_2_), structural components (Ba_3_-Ba_5_, Fu_3_-Fu_5_, OP_2_-OP_5_), respectively; υ(i) is the c-*p* value of the i-th taxon of non-plant parasites or plant parasites; and f(i) is the proportion of the number of individuals in the i-th taxon to the total number of individuals, respectively.

### 4.4. Soil Organic Matter

The method proposed by Cambardella and Elliott [77] was used to separate particulate organic carbon (POC) and mineral-associated organic carbon (MAOC). The procedure involves taking 15 g of an air-dried soil sample from each plot, adding 60 mL of 5 g/L sodium hexametaphosphate, and shaking at a speed of 90 rpm for 18 h on a reciprocal shaker. The soil suspensions were passed through a 53 μm sieve and rinsed several times with water. The material retained on the sieve (POM) and the material passed through the sieve (MAOM) were collected and dried at 60 °C to constant mass, and their percentages of the total soil mass were calculated. After grinding, the samples were passed through a 100-mesh sieve, and the organic carbon content of each component was measured using an automatic elemental analyzer. The POC and MAOC were calculated based on the carbon content of each organic component and their mass percentage.

### 4.5. Data Analysis

The lmer function of the “lmerTest” package was used to construct a linear mixed model, with PFG removal set as a fixed effect while block was set as the random effect to evaluate the effects of PFG removal on root properties, soil properties, nematode abundance, and nematode indices. The glht function with Tukey method of the “multcomp” package was used for multiple comparisons to test the differences among the PFGs removal treatments [78]. Correlations between different nematode trophic groups and plant and soil environmental variables were performed by Mantel tests using the “vegan” package [79]. Graphs were drawn using the ggplot function in the “ggplot2” package [80]. The data in the graphs were presented as mean and standard error (n = 5). Data analysis and visualization were performed using R software version 4.2.2 [81].

## 5. Conclusions and Outlook

In this study, we showed that PFGs significantly modified nematode abundance and community structure and soil carbon fractions. We also revealed a positive correlation between soil organic carbon and the abundance of bacterivores, indicating that bacterivores may play a significant role in organic carbon cycling. However, other nematode trophic groups showed no significant response to soil carbon fractions, suggesting that while high organic carbon is essential for the nematode community, other primary limiting factors persist in alpine meadow ecosystems with higher organic matter content, warranting further study. Our findings underscore that PFG removal alters nematode community and carbon fractions through changes in resource quality and quantity, highlighting the imperative of preserving or restoring PFG diversity to maintain soil carbon sequestration and ecosystem stability.

## Figures and Tables

**Table 1:** Results of linear mixed model of root and soil properties under different PFGs treatments.

Index	*F*	*p*
**Root properties**		
Root biomass	**4.716**	**0.006**
Root carbon	0.253	0.905
Root nitrogen	0.704	0.598
Root carbon to nitrogen ratio	0.730	0.580
**Soil properties**		
Soil water content	0.999	0.427
Soil organic carbon	0.527	0.717
Soil nitrogen	0.615	0.657
Soil carbon to nitrogen ratio	1.181	0.348
MAOC	0.122	0.973
POC	**14.972**	**0.000**

MAOC: Mineral-associated organic carbon, POC: Particulate organic carbon. Values are in bold when *p* <  0.05.

**Table 2:** Results of linear mixed model of nematode characteristics under different PFG treatments.

Index	*F*	*p*
Nematode abundance		
Total abundance	**4.395**	**0.011**
Bacterivores abundance	2.337	0.090
Fungivores abundance	1.750	0.179
Plant parasites	**4.719**	**0.008**
Omnivores-predators	**4.747**	**0.008**
Nematode indices		
Shannon–Wiener index	**3.321**	**0.031**
Nematode channel ratio	0.487	0.745
Channel index	2.569	0.069
Enrichment index	0.360	0.834
Structure index	**5.933**	**0.002**
Free-living nematode maturity index	**27.475**	**0.000**
Plant parasite index	**7.392**	**0.000**

Values are in bold when *p* <  0.05.

**Table 3:** Relative abundance of nematodes under different PFG treatments.

Genera	Trophic Group	Treatments				
		CK	Forbs	Graminoids	Legumes	Removal All
*Acrobeloides*	Ba_2_	5.51 ± 1.14	5.40 ± 0.49	7.37 ± 1.59	4.87 ± 1.70	7.00 ± 1.97
*Cephalobus*	Ba_2_	3.77 ± 1.60	2.81 ± 0.51	4.75 ± 2.12	2.02 ± 1.07	1.27 ± 0.68
*Plectus*	Ba_2_	4.87 ± 1.12	1.90 ± 0.68	3.94 ± 0.87	1.82 ± 0.77	1.82 ± 0.95
*Alaimus*	Ba_4_	3.86 ± 1.08	2.83 ± 0.86	1.02 ± 0.39	3.25 ± 1.17	2.06 ± 0.78
*Eucephalobus*	Ba_2_	0.78 ± 0.37	0.74 ± 0.53	1.81 ± 0.64	1.90 ± 0.54	2.50 ± 0.96
*Mesorhabditis*	Ba_1_	0	0.20 ± 0.20	2.54 ± 1.43	0.58 ± 0.38	0.36 ± 0.22
*Anaplectus*	Ba_2_	0.20 ± 0.20	0.19 ± 0.19	0.60 ± 0.38	0.88 ± 0.47	0.99 ± 0.79
*Panagrolaimus*	Ba_1_	0.95 ± 0.52	0.36 ± 0.36	0	0.69 ± 0.50	0.79 ± 0.48
*Wilsonema*	Ba_2_	0.20 ± 0.20	0.58 ± 0.39	0.55 ± 0.36	0.37 ± 0.23	0.18 ± 0.18
*Protorhabditis*	Ba_1_	0.39 ± 0.24	0.79 ± 0.79	0.36 ± 0.22	0	0.18 ± 0.18
*Teratocephalus*	Ba_3_	0.19 ± 0.19	0.18 ± 0.18	0.37 ± 0.22	0.20 ± 0.20	0.18 ± 0.18
*Diplogaster*	Ba_1_	0.76 ± 0.36	0	0	0	0
*Amphidelus*	Ba_4_	0.20 ± 0.20	0	0	0.20 ± 0.20	0
*Bastiania*	Ba_3_	0	0	0	0	0.18 ± 0.18
*Rhabditis*	Ba_1_	0	0	0.18 ± 0.18	0	0
*Filenchus*	Fu_2_	7.63 ± 3.15	5.41 ± 0.88	11.25 ± 2.42	3.75 ± 1.82	3.78 ± 1.03
*Tylencholaimus*	Fu_4_	3.99 ± 1.37	3.91 ± 1.13	5.10 ± 0.85	7.31 ± 3.67	5.49 ± 1.64
*Leptonchus*	Fu_4_	0.20 ± 0.20	0.20 ± 0.20	0	0	0.18 ± 0.18
*Helicotylenchus*	PP_3_	13.39 ± 4.42	27.46 ± 3.24	19.26 ± 2.46	11.39 ± 2.51	12.11 ± 3.38
*Pratylenchus*	PP_3_	2.48 ± 0.77	2.25 ± 0.75	1.27 ± 0.54	5.81 ± 3.69	4.33 ± 1.44
*Tylenchus*	PP_2_	0.38 ± 0.24	0	0.78 ± 0.53	0.76 ± 0.37	0.75 ± 0.55
*Tylenchorhynchus*	PP_4_	0	0	0	0	0.41 ± 0.41
*Aporcelaimus*	OP_5_	18.96 ± 1.82	16.99 ± 3.26	11.38 ± 3.19	16.36 ± 3.44	19.24 ± 1.92
*Eudorylaimus*	OP_4_	12.74 ± 1.65	9.39 ± 2.33	11.36 ± 4.10	12.34 ± 2.89	17.17 ± 4.35
*Discolaimus*	OP_4_	3.85 ± 1.12	7.82 ± 2.21	3.97 ± 1.26	6.53 ± 2.21	7.42 ± 3.74
*Epidorylaimus*	OP_4_	1.93 ± 1.03	4.12 ± 0.65	1.70 ± 0.66	7.18 ± 4.63	3.91 ± 1.14
*Prodorylaimus*	OP_4_	5.47 ± 2.58	1.31 ± 0.55	1.08 ± 0.72	6.06 ± 4.48	2.21 ± 1.18
*Dorylaimellus*	OP_5_	3.48 ± 2.78	2.59 ± 1.41	3.45 ± 0.33	2.31 ± 1.00	3.24 ± 1.72
*Mononchus*	OP_4_	0.57 ± 0.23	0.37 ± 0.23	2.01 ± 0.93	0.89 ± 0.47	0.37 ± 0.22
*Mesodorylaimus*	OP_4_	1.16 ± 0.57	0.36 ± 0.22	1.10 ± 0.67	0.58 ± 0.40	0.73 ± 0.53
*Microdorylaimus*	OP_4_	0.96 ± 0.43	1.09 ± 0.67	0.74 ± 0.54	0.19 ± 0.19	0.18 ± 0.18
*Prionchulus*	OP_4_	0.57 ± 0.23	0.18 ± 0.18	1.64 ± 0.83	0.19 ± 0.19	0.38 ± 0.38
*Tripyla*	OP_3_	0.36 ± 0.36	0.18 ± 0.18	0.42 ± 0.26	0.18 ± 0.18	0.41 ± 0.41
*Mylonchulus*	OP_4_	0.20 ± 0.20	0.20 ± 0.20	0	0.88 ± 0.47	0.18 ± 0.18
*Axonchium*	OP_5_	0	0	0	0.51 ± 0.51	0
*Enchodelus*	OP_4_	0	0.19 ± 0.19	0	0	0

The numbers in each trophic group indicate the c-*p* value (e.g., Ba1 represents bacterivores with a c-*p* value of 1). Dominant groups (>10%); Common groups (1–10%); Rare groups (<1%).

## Data Availability

The original contributions presented in this study are included in the article/Supplementary Material. Further inquiries can be directed to the corresponding authors.

## Supplementary Materials

The following supporting information can be downloaded at: https://www.mdpi.com/article/10.3390/plants14243728/s1, Table S1. Ecological index of nematode under different PFGs treatments. Table S2. Soil properties and root properties under different PFGs treatments.
