# Supplementary material for: Plant Functional Group Removal Shifts Soil Nematode Community and Decreases Soil Particulate Organic Carbon in an Alpine Meadow
Source: Plants (Basel). 2025 Dec 6;14(24):3728. doi: 10.3390/plants14243728 (PMC12737263; doi:10.3390/plants14243728)
Supplement: Supplementary file 1 [file plants-14-03728-s001.zip › plants-3995461-supplementary.pdf]

plants

MDPI

Supplementary File

Table S1. Ecological index of nematode under different PFGs treatments.

Index	CK	Forbs	Graminoids	Legumes	All-plants-removed
H'	2.46 ± 0.09 ab	2.29 ± 0.12 b	2.56 ± 0.03 a	2.35 ± 0.05 ab	2.37 ± 0.07 ab
NCR	0.65 ± 0.06 a	0.62 ± 0.04 a	0.56 ± 0.06 a	0.65 ± 0.11 a	0.66 ± 0.05 a
EI	38.38 ± 6.79 a	33.24 ± 7.57 a	35.17 ± 3.10 a	34.90 ± 5.42 a	33.26 ± 3.10 a
SI	91.25 ± 1.65 a	92.91 ± 0.54 a	84.02 ± 4.05 b	94.36 ± 0.49 a	93.88 ± 0.46 a
CI	52.85 ± 15.69 ab	75.20 ± 15.90 a	62.67 ± 2.92 ab	41.78 ± 17.13 ab	30.87 ± 3.75 b
MI	3.19 ± 0.09 a	2.69 ± 0. 06 b	2.59 ± 0.06 b	3.12 ± 0.08 a	3.30 ± 0.04 a
PPI	0.48 ± 0.15 b	0.87 ± 0.02 a	0.55 ± 0.01 b	0.53 ± 0.06 b	0.40 ± 0.04 b

H': Shannon - Wiener index, NCR: Nematode channel ratio; EI: Enrichment index, SI: Structure index, CI: Channel index, MI: Free-living nematode maturity index, PPI: Plant parasite index. Data are means ± standard errors, n = 5.

Table S2. Soil properties and root properties under different PFGs treatments.

Index	CK	Forbs	Graminoids	Legumes	All-plants-removed
Root C (g kg^{-1})	422.3 ±16.2 a	424.6 ± 13.8 a	420.3 ±13.6 a	423.5 ±10.2 a	409.1 ±15.4 a
Root N (g kg^{-1})	10.71 ± 1.34 a	11.30 ± 1.11 a	10.84 ± 0.79 a	11.37 ±0.94 a	12.52 ± 0.48 a
Root C/N	42.78 ±6.95 a	39.41 ± 4.91 a	39.98 ± 4.13 a	38.62 ± 4.21 a	32.93 ± 2.04 a
Root biomass (g)	1.41 ± 0.30 b	1.68 ± 0.05 ab	2.22 ± 0.18 a	1.81 ±0.28 ab	1.07 ± 0.28 b
SWC (%)	39.08 ± 1.17 a	37.31 ± 1.57 a	36.96 ± 2.24 a	36.21 ± 1.09 a	35.34 ± 1.46 a
SOC (g kg^{-1})	57.72 ± 2.76 a	51.69 ± 0.52 a	55.53 ± 7.16 a	52.13 ± 1.89 a	49.64 ± 3.70 a
Soil N (g kg^{-1})	5.14 ± 0.19 a	4.64 ± 0.05 a	4.96 ± 0.58 a	4.75 ± 0.10 a	4.52 ± 0.33 a
Soil C/N	11.21 ± 0.19 a	11.14 ± 0.01 a	11.13 ± 0.15 a	11.34 ± 0.19 a	10.97 ± 0. 12 a
MAOC (g kg^{-1})	6.06 ± 0.82 a	6.23 ± 1.03 a	6.29 ± 1.00 a	7.12 ± 0.99 a	5.38 ± 0.13 a
POC (g kg^{-1})	33.42 ± 2.08 a	37.74 ± 1.78 a	24.76 ± 1.79 b	29.06 ± 1.52 b	22.91 ± 1.60 b

Root C: Root carbon, Root N: Root nitrogen, Root C/N: Root carbon to nitrogen ratio, SWC: Soil water content, SOC: Soil organic carbon, Soil N: Soil nitrogen, Soil C/N: Soil carbon to nitrogen ratio, MAOC: Mineral-associated organic carbon, POC: Particulate organic carbon. Data are means ± standard errors, n = 5.
